# Protocol for isolation and expansion of natural killer cells from human peripheral blood scalable for clinical applications

**DOI:** 10.1093/biomethods/bpaf015

**Published:** 2025-02-26

**Authors:** Soumyadipta Kundu, Leonie Durkan, Michael O’Dwyer, Eva Szegezdi

**Affiliations:** School of Biological and Chemical Sciences, University of Galway, Galway, H91 TK33, Ireland; School of Biological and Chemical Sciences, University of Galway, Galway, H91 TK33, Ireland; School of Medicine, University of Galway, Galway, H91 TK33, Ireland; School of Biological and Chemical Sciences, University of Galway, Galway, H91 TK33, Ireland

**Keywords:** NK cell isolation, NK cell expansion, haematological malignancies, cancer cellular immunotherapy

## Abstract

Natural killer (NK) cells have emerged as promising candidates for novel immunotherapy strategies against various malignancies. Their unique ability to recognize and eliminate tumour cells without prior sensitization, coupled with the secretion of pro-inflammatory cytokines such as interferon-gamma and tumour necrosis factor, position them as promising agents in cancer therapy. Adoptive NK cell transfer has shown particular promise in haematological malignancies, where NK cell infusions could achieve remission in a high proportion of patients. Moreover, the possibility to engineer NK cells to express chimeric antigen receptors can further enhance their efficacy, thereby broadening their applicability to include solid tumours. Ongoing research is crucial to optimize NK cell therapies and enhance their efficacy to expand their clinical applications. However, this research hinges on robust protocols and experimental methodology for the isolation, expansion, and genetic engineering of NK cells. In an attempt to set up a standardized protocol for NK cell isolation and expansion, we present a thoroughly tested and validated protocol that can produce highly pure, viable, and potent NK cells that can be used for research and development of NK cell therapies. The protocol is highly reproducible, closely aligned to comply with Good Manufacturing Practice regulations, and tested for scalability to produce NK cells at clinically relevant dosages to support the development of off-the-shelf NK products.

## Introduction

Natural killer (NK) cells are a crucial component of the innate immune system, recognized for their ability to identify and eliminate infected and malignant cells without requiring prior sensitization to specific antigens. Unlike T and B cells, which use antigen-specific receptors (e.g., T-cell receptor or B-cell receptor) for identification of malignant cells, NK cells use a variety of pattern-recognition receptors to detect targets. These receptors recognize a range of antigens, including stress-induced molecules that are often up-regulated on malignant cells. For instance, the NKG2D receptor, belonging to the killer lectin-like receptor family, recognizes ligands which are frequently expressed on tumour cells undergoing oncogenic stress, such as major histocompatibility complex (MHC) class I chain-related protein A (MIC-A) and MHC class I chain-related protein B (MIC-B) and UL16-bindingproteins (ULBPs) [1, 2]. NKp46, another NK cell receptor under the natural cytotoxicity receptor family, has been shown to bind to surface-expressed calreticulin (ecto-CRT), a marker of endoplasmic reticulum stress, which is also commonly observed on cancer cells [3].

The activity of NK cells is kept in check by receptors that recognize self MHC-I molecules. Healthy cells typically express normal levels of MHC class I molecules, which are recognized by NK inhibitory receptors, such as the killer immunoglobulin-like receptors (KIRs) and CD94/NKG2A. This interaction signals NK cells to recognize these cells as ‘self’ and be spared, while cells with reduced or no MHC-I expression, typical of malignant cells, are flagged as potential targets for NK cell-mediated cytotoxicity. Contrasting to NK cells, tumour cell recognition by cytotoxic T cells depends on the presentation of tumour-associated antigens by MHC-I. However, many cancer cells evade this adaptive immune response by down-regulating MHC expression, rendering them ‘invisible’ to cytotoxic T cells. Hence, NK cells can effectively complement the T cells in addressing this immune evasion strategy and thus positioning them as a focal point in current cancer immunotherapy research.

The application of NK cells as an adoptive cell therapy also exhibits a more favourable safety profile compared to T-cell therapies, with lower risk of severe side effects, such as cytokine release syndrome and graft-versus-host disease (GvHD) [4, 5]. Due to their higher safety profile, NK cells can be used not only as an autologous treatment like chimeric antigen receptor (CAR)-T therapies [6], but also as an allogeneic therapy. This allogeneic nature also allows for the possibility of developing ‘off-the-shelf’ therapies, providing a more accessible treatment option for patients.

Although NK cells show great potential in cancer immunotherapy, several limitations hinder their clinical use. The choice of isolation method is critical for the generation and testing of cell therapy products, where preserving the viability, purity, immunophenotype and functionality of the cell product is of paramount importance [7]. However, with no currently approved NK cell therapy products, there is significant variation in the methods used to isolate and expand NK cells (Table 1). Studies comparing the performance of NK cells isolated using a fully automated continuous centrifugal microfluidics system against those obtained through manual methods showed that NK cells isolated with the automated method exhibited superior proliferation and cytolytic efficacy, suggesting that minimizing cell stress during isolation is important to retain the function of NK cells [12]. Newly developed microfluidic systems are able to reduce stress during the isolation process, improving NK cell function, but they have a high operational cost which can be prohibitive for most translational research studies. Conventional flow cytometric sorting, though less expensive and fairly precise, exposes the NK cells to mechanical stress and prolonged handling times, which can compromise their viability and functionality [13]. Alternatively, although magnetic immunolabeling effectively separates NK cells, depending on the method used, can alter the expression of NK cell surface markers. This can further affect their responsiveness and cytotoxicity, which would ultimately impact their therapeutic potential [14]. Comparative analysis using various cell isolation methods revealed that negative selection methods resulted in higher purity and yield of NK cells in a shorter time frame, which is crucial for maintaining their functional characteristics [15].

**Table 1.** Commonly used natural killer cell isolation methods.

Isolation method	**Purity achieved**, %	Reference
EasySep Human NK Cell Enrichment Kit—immunomagnetic negative selection to deplete non-NK cells from PBMC fraction	89	[8]
NK isolation kit—negative immunomagnetic isolation to deplete non-NK cells from PBMC fraction	92.68 ± 2	[9]
Magnetic CD3^+^ depletion followed by CD56^+^ selection	Not reported	[10]
RosetteSep—negative isolation to deplete non-NK cells from PBMC fraction	≥95	[11]
Flow cytometric cell sorting to (CD56+/CD3−)	≥99	[11]
*Current protocol: NK isolation kit—negative isolation to deplete non-NK cells from PBMC fraction*	*97–99*	*N/A*

Thus, optimizing the manufacturing process for NK cells to ensure consistent quantity, quality, and functionality is needed to facilitate their better translation from research to clinical applications, and to ultimately improve patient outcomes [16, 17].

Once isolated, NK cells require *ex vivo* expansion to reach sufficient quantities for therapeutic doses. Similar to the isolation techniques, NK cell expansion protocols show considerable variation (Table 2). Methods typically employ the use of different cytokines including interleukin (IL)-2, IL-21, IL-15, IL-18, alone or in varying combinations [22, 23]. Higher expansion numbers are achieved by the use of irradiated feeder cells such as the now clinically approved Epstein-Barr virus transformed lymphoblastoid cell lines (EBV-LCL) or K562 cells engineered to express membrane bound IL-21 and 4-1BB ligand (K562.mbIL21.4-1BBL) [24–26]. The use of both soluble or membrane bound IL-21 expressing feeder cells has also been reported to achieve significant *ex vivo* expansion of NK cells. NK cells expanded under these conditions exhibited potent cytotoxic activity against cancer cells *in vitro* and maintained this activity when tested in xenograft mouse models. Moreover, the findings indicate that IL-15, while previously noted for its role in NK cell expansion, may not be as effective as IL-21 in promoting long-term persistence of NK cells *in vivo*, indicating the importance of cytokine signalling in shaping the immune response and enhancing the therapeutic applications of NK cells [27, 20].

**Table 2.** Manual natural killer cell expansion methods.

Expansion method		Average fold expansion	Reference
Non-feeder cell based	500 IU IL-2	7.5-fold by 2 weeks	[14]
	500 IU IL-2	14 ± 13-fold by 2 weeks	[10]
	1000 IU IL-2 (MACS medium)	24.2–45.9-fold by 2 weeks	[18]
	Stimulation with 10 ng/ml IL-15, then second stimulation with 25 ng/ml IL-21 three days before analysis	4.5-fold by day 10	[8]
Feeder cell based	CD3-depleted PBMCs stimulated with 50 IU/ml IL-2 + K562 Clone9.mbIL21 feeder cells (2:1, feeder:PBMC). Feeder cells refreshed every 7 days (1:1).	47,967 ± 42,230 by 3 weeks	[19]
	CD3-depleted PBMCs stimulated with 50 IU/ml IL-2 + K562 Clone9.mbIL15 feeder cells (2:1, feeder:PBMC). Feeder cells refreshed every 7 days (1:1).	825 ± 1108 by 3 weeks	[19]
	OCI-AML3 feeder cells transduced with mbIL-21 + 200 IU/ml IL-2. Feeder cells added weekly (5:1, feeder:NK)	Approx. 700 ± 245-fold by 3 weeks	[20]
	IL-2 500 IU/ml and EBV-LCL (20:1, feeder:NK)	1344 ± 1135-fold by 2 weeks 815–3267-fold by day 16	[10] [21]
	*Current protocol: Stimulation with 20 ng/ml IL-21 + 100 IU/ml IL-2 and EBV-LCL feeder cells (10:1, feeder:NK) on day 0. IL-2 replenished every 2–3 days.*	*289 *±* *70*-fold by 2 weeks* *10,460 *±* *4972 *fold by 3 weeks*	*N/A*

Translating NK cell research for successful clinical application would be facilitated by robust and reproducible protocols for isolation and expansion of NK cells. Previously, we have discussed various parameters to be considered for the generation of NK cells for adoptive cell transfer and the characteristics of an ideal cell expansion platform [28]. Here, we present an optimized, thoroughly tested and reproducible protocol that can be used to isolate and expand NK cells from peripheral blood to achieve pure, viable, and highly functional NK cells. This method provides an ideal foundation for producing NK cells for a broad range of research applications. In addition, this method is designed with an outlook for scalability and translation for clinical applications.

## Materials and methods

### Preparation work prior to NK cell isolation

#### Generation of feeder cells

Epstein Barr Virus-transformed lymphoblastoid (EBV-LCL) feeder cells, naturally expressing ligands relevant to NK cell activation and proliferation receptors such as 4-1BB, CD48, and CD58 were obtained from Dr Richard Childs of the National Institutes of Health (NIH). The cells can be expanded to large quantities using a 100M G-Rex culture flask or alternatively, the feeder cells can be expanded in T25, T75, or T175 culture flasks until sufficient cell numbers are obtained. For expansion using a 100M G-Rex culture flask, 50 x 10^6^ EBV-LCL cells are seeded into the flask in 1000 ml complete Roswell Park Memorial Institute (RPMI) 1640 medium supplemented with 10% foetal bovine serum (FBS), Pen (100 U/ml)/Strep (100 µg/ml), 1 mM sodium pyruvate and 1X RPMI non-essential amino acids and are incubated for 10 days at 37°C, 5% CO_2_ atmosphere. Change 75% of the medium on the seventh day without disturbing the cell layer at the bottom of the flask. Cells are ready to be harvested by day 14. A confluent, white layer of cells will cover the base of the flask. To collect the cells, remove as much culture medium as possible without disturbing the cells. Mix the cells gently by pipetting then collect and divide the cell suspension into required number of 50 ml tubes. Centrifuge at 300 x g for 5 minutes. Re-suspend the cell pellets in 5 ml of complete RPMI medium and pool the cells together into one 50 ml tube. Top up to 40 ml total volume with RPMI medium and perform a cell count. Take 1 x 10^9^ EBV-LCL cells for irradiation. Expose cells to 100 Grey gamma-irradiation to arrest their proliferative ability. Following irradiation, aliquot the cells into cryovials at 25 x 10^6^ cells/vial in complete RMPI medium containing 10% dimethyl sulfoxide (DMSO). Following rate-controlled freezing, transfer aliquots to liquid nitrogen for long-term storage. Use the remaining, non-irradiated EBV-LCL cells to prepare a cell bank for future expansion as needed. Centrifuge the cells at 300 x g for 5 minutes. Discard the supernatant. Re-suspend in complete RPMI with 10% DMSO and divide into cryovials at 10 x 10^6^ cells/vial for controlled rate freezing. After 24 h transfer aliquots to liquid nitrogen. Material vendors and specific catalogue numbers are provided in Table 3.

**Table 3.** List of required materials for natural killer cell isolation, expansion, and cryopreservation.

Material	Vendor	Catalogue number
Dulbecco′s PBS	Sigma-Aldrich	D8537
Ficoll-Paque premium	Sigma-Aldrich	GE17-5442-03
0.5 M EDTA disodium salt solution	Sigma-Aldrich	03690
BSA	Sigma-Aldrich	A9647
hAB serum	Sigma-Aldrich	H3667
NK MACS media and supplement	Miltenyi Biotec	130-114-429
IL-2	Miltenyi Biotec	130-097-743
IL-21	Miltenyi Biotec	130-094-563
NK cell isolation kit human	Miltenyi Biotec	130-092-657
LS columns	Miltenyi Biotec	130-042-401
MACS multi-stand	Miltenyi Biotec	130-042-303
Midi MACS separator	Miltenyi Biotec	130-042-302
CryoStor^®^ CS10	Biolife Solutions	210102
Benzonase	Sigma-Aldrich	E1014
EBV-LCL cell line, irradiated post expansion	NIH	NA
RPMI 1640 medium	ThermoFisher Scientific	61870010
Sodium pyruvate	Sigma-Aldrich	S8636
RPMI 1640 amino acids solution	Sigma-Aldrich	R7131
Optional: G-Rex^®^100M bioreactor	Wilson Wolf	81100
Transfer pipette	Sarstedt	861172010
Serological pipette 25 ml	Sarstedt	861685001
Serological pipette 10 ml	Sarstedt	861254001
Serological pipette 5 ml	Sarstedt	861253001
T25 tissue culture flask	Sarstedt	833910002
T75 tissue culture flask	Sarstedt	833911002
SepMate™-50 tubes	StemCell Technologies	85450
BD vacutainer K2-EDTA blood collection tubes	Fisher Scientific	10331254
Blood samples from healthy donors		

#### Culture of chronic myelogenous leukaemia cell line K562 cells

K562 cells have attained widespread use as a highly sensitive *in vitro* target for testing NK cell cytotoxicity. They can be cultured in complete RPMI medium as mentioned in the preceding section. Cells should be seeded at a density of 2 x 10^5^ cells/ml and subcultured once the count is in the range of 1 to 2 x 10^6^ cells/ml in standard tissue culture flasks, every 2–3 days approximately.

##### NK cell medium and buffer preparation

Complete NK MACS medium is composed of basal NK MACS medium supplemented with 1% (v/v) NK MACS supplement and 5% (v/v) heat inactivated human AB (hAB) serum. To prepare MACS buffer, dissolve 2.5 g of bovine serum albumin (BSA) into 500 ml phosphate buffered saline (PBS). Add 2 ml of 0.5 M ethylenediaminetetraacetic acid (EDTA) and filter-sterilize the solution through a 0.2 µm filter. Store the buffer at 2–8°C for up to three months.

#### NK cell isolation

**Figure 1.**
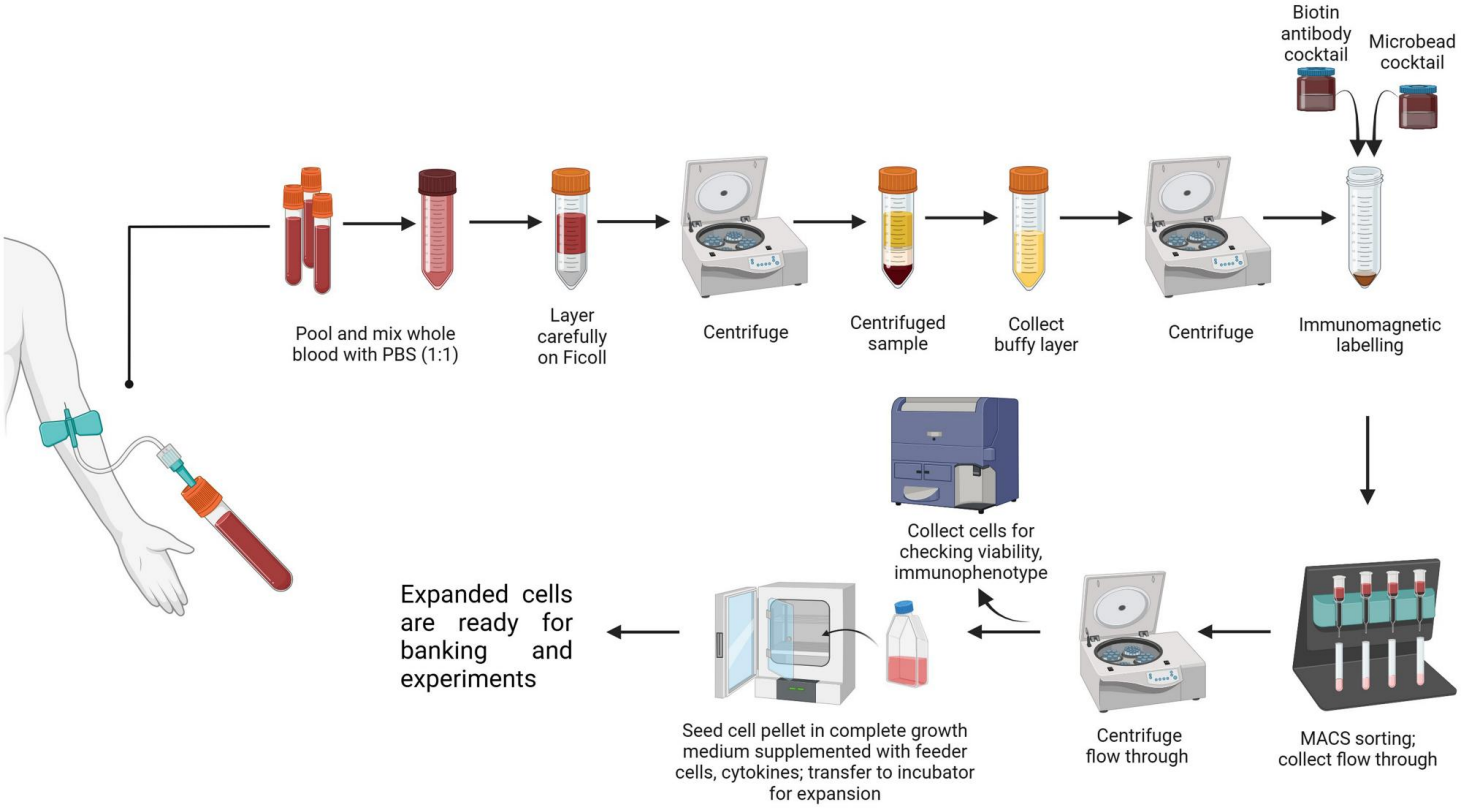
Graphical representation of the workflow of natural killer cell isolation from peripheral blood. The protocol takes in total 3 hours of work to generate NK cells ready for expansion. Blood is collected from healthy donors and the peripheral blood mononuclear cell fraction is collected as the buffy layer following Ficoll gradient centrifugation. The mononuclear cells are incubated with biotin-conjugated antibodies against non-NK cell type-specific surface markers and a superparamagnetic biotin-binding microbead cocktail. The cell suspension is then passed through a MACS column on a magnetic MACS separator. The NK cells are isolated by negative magnetic selection whereby they flow though the column unlabelled, while the labelled non-NK cell types are retained in the column. The NK cells are then cultured with irradiated feeder cells in the presence of cytokines for subsequent expansion. Figure was created using BioRender.com

An illustration to summarise the method workflow is provided in Fig. 1. All work with blood and NK cell cultures should be carried out in a class 2 biological safety cabinet using aseptic techniques.

**Figure 2.**
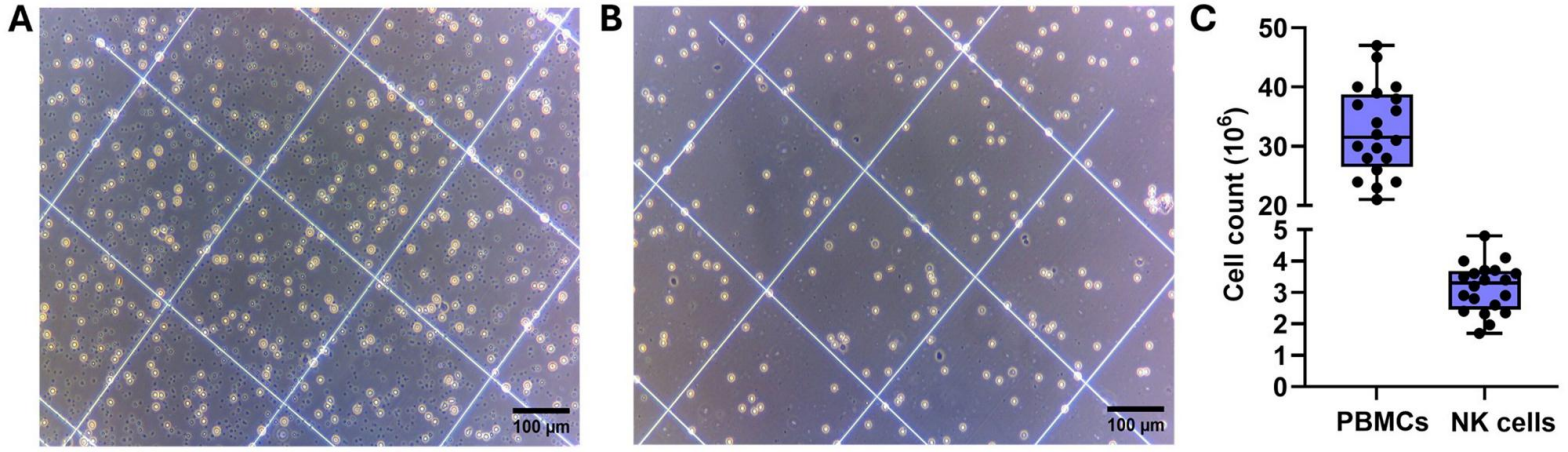
Representative images of cell fractions collected during the cell counting steps of the isolation protocol. Expected number of NK cells that can be obtained after isolation. (A) Total PBMC fraction prior to magnetic sorting. Platelets are also visible as smaller, darker spots in the image. (B) NK cells collected in the flow-through after negative MACS selection appear similar to other haematopoietic cells as small, round, and bright. Background debris from platelets is reduced. Images were taken under 100x magnification. Scale bars show 100 μm. (C) Number of total PBMCs and the number of NK cells harvested from 25 ml peripheral blood (*n* = 20)

*Isolation of mononuclear cells*. Collect 25 ml of blood from healthy donors in vacutainer tubes (EDTA-coated). Pool blood from a single donor together in a 50 ml tube and add sterile PBS to obtain a 1:1 dilution. Aliquot 30 ml of Ficoll into two 50 ml SepMate™ tubes, 15 ml each. Gently vortex the blood and PBS mixture and transfer 25 ml of the homogeneous PBS diluted blood to each of the 50 ml tubes by gently layering it over the Ficoll at slow speed. Final volume per tube is 40 ml. Centrifuge the two tubes at 700 x g for 10 minutes, six acceleration, six deceleration. Remove the buffy layer using a transfer pipette, and transfer into a new 50 ml tube containing 20 ml of PBS. Centrifuge the tube at 300 x g for 5 minutes, maximum acceleration and deceleration. Discard the supernatant and re-suspend cells in 10 ml of cold MACS buffer. Take 10 µl of the cell suspension for counting using a traditional haemocytometer. See Fig. 2A for a representative image of cells at this stage.

Count and calculate the total cell number as follows:

(____cells/16-square quadrant) *10^4^* 10 ml = ____).

Total cell number will vary between donors. Convert total mononuclear cell number to *__ x10^7^* format to permit calculations for next steps.

**Note:** As an alternative to SepMate™ tubes, the Ficoll separation can be carried out using 50 ml Falcon tubes. Using the same volumes as above but a longer centrifugation step at 700 x g for 20 minutes, one acceleration, zero deceleration (Supplementary data, Video 1).

*Magnetic labelling*. Centrifuge the cells at 300 x g for 5 minutes. Discard the supernatant completely. Re-suspend cells in 40 µl of MACS buffer per 10^7^ cells. Add 10 µl of Biotin-antibody cocktail/10^7^ cells from the NK cell isolation kit. Mix well and incubate for 10 minutes at 2–8°C in the refrigerator, not on ice, as per manufacturer’s instructions. After incubation, add 30 µl of MACS buffer/10^7^ cells and 20 µl of the microbead cocktail/10^7^ cells. Mix well and incubate again for 10 minutes at 2–8°C. Finally, add an appropriate volume of MACS buffer to make up to a final volume of 1000 µl. A worked example is provided in Table 4.

**Table 4.** A worked example of volume calculations for magnetic labelling and isolation of natural killer cells from mononuclear cells.

	Donor 1
Total mononuclear cell count	(190 cells/quadrant) *10^4^*10 ml = 1.9 x 10^7^ total cells
MACS buffer to add (40 µl/10^7^ cells)	76 µl
Biotin antibody cocktail to add (10 µl/10^7^ cells)	19 µl
*10 min incubation at 2–8* ^o^C	
MACS buffer to add (30 µl/10^7^ cells)	57 µl
Microbead cocktail to add (20 µl/10^7^ cells)	38 µl
*10 min incubation at 2–8* ^o^C	
MACS buffer to make up to 1000 µl total volume	810 µl
Proceed with negative magnetic separation through LS column	
Final count of isolated NK cells post-magnetic separation	(170 cells/quadrant) *10^4^* 1 ml = 1.7 x 10^6^ NK cells

*Cell separation*. Place an LS column into the Midi MACS separator magnet that has been attached to the MACS multi-stand. One column is used per donor and has the capacity for up to 100 x 10^6^ labelled cells or 2 x 10^9^ total cells. Prepare the column by rinsing with 3 ml of MACS buffer. Discard the flow-through then place a sterile 15 ml tube beneath the column in which to collect the NK cells.

Apply the 1000 µl of cell suspension from the previous section to the LS column. Allow cells to flow through the column and down into the 15 ml tube. Wash the column twice with 3 ml MACS buffer, continuing to collect the flow through. Centrifuge the tube of isolated cells at 300 x g for 5 minutes. Discard the supernatant and re-suspend the cells in 1 ml of complete NK MACS medium.

Count cells as before.

Total cell number will vary between donors, but the expected yield from 25 ml of peripheral blood is approximately 1.5–3.5 x 10^6^ NK cells.

If required, cell sample can be collected at his stage for testing NK cell viability and immunophenotype. This is described in the section ‘Immunophenotyping of NK cells’.

**Note:** If you wish to collect the non-NK cell fraction that is retained in the column, remove the column from the magnetic separator, place into a new sterile 15 ml tube. Pipette 5 ml of MACS buffer onto the column and flush the cells down using the plunger that is supplied with the column.

### NK cell seeding and feeder cell addition

The total culture volume is 20 ml per T75 flask with a staring NK cell quantity of 1 x 10^6^ cells/flask. First prepare the irradiated EBV-LCL feeder cells. Remove the feeder cells from liquid nitrogen and quickly thaw in a water bath. Transfer the cells into 5 ml of PBS and collect them by centrifuging at 300 x g for 5 minutes. Discard the supernatant and re-suspend the EBV-LCL cells in 2.5 ml NK MACS medium to create a density of 10 x 10^6^ cells/ml (feeder cells were frozen down at 25 x 10^6^ per vial, as mentioned above). Seed 1 x 10^6^ NK cells in complete NK MACS medium per T75 flask. Add 10 x 10^6^ irradiated EBV-LCL cell suspension (1 ml) to the flask of NK cells to obtain a 1:10 ratio of NK: Feeder cells. Top up the flasks with complete NK medium to a final volume of 20 ml. Add IL-21 to a final concentration of 20 ng/ml and add IL-2 at 500 IU/ml. Incubate the cells at 37°C, keeping the flasks standing upright. Add fresh IL-2 at 500 IU/ml to the cell cultures after 2 days. On day four, collect the cells into a 50 ml tube. Centrifuge at 300 x g for 5 minutes. Then discard 15 ml of the supernatant and replace with 5 ml complete NK MACS medium. Count the cells and re-seed at a density of 0.5 x 10^6^ cells/ml in up to 8 ml in T25 culture flasks, or, up to 20 ml in T75 culture flasks and supplement with fresh IL-2 at 500 IU/ml. For re-seeding cells from day 4 onwards, keep culture flasks lying horizontal inside the incubator. Continue to subculture the NK cells every 2–3 days to maintain a density of 0.5 x 10^6^ cells/ml, supplementing with 500 IU/ml of IL-2 each time.

**Note:** This method has also been tested on the Good Manufacturing Practice (GMP) grade G-Rex^®^100M platforms to test scalability. Starting volume may be increased to 50–60 ml peripheral blood to ensure that a minimum starting amount of 4–5 x 10^6^ NK cells are obtained. NK cells can be isolated using the same protocol as above and seeded in G-Rex^®^100M flasks along with irradiated EBV-LCL feeder cells at 1:10 (NK: Feeder cell) ratio. The flasks can be topped-up with 1000 ml of complete NK MACS medium, 500 IU/ml IL-2 (Premium grade, Miltenyi Biotec, #130-097-745) and 20 ng/ml IL-21 (Premium grade, Miltenyi Biotec, #130-095-769). Add 500 IU/ml of IL-2 from Day 2 onwards and then every 48 h.

### Immunophenotyping of NK cells

Purity of the isolated NK cells is of utmost importance for all downstream research. Hence, we optimized a flow cytometry panel to check the viability and purity of the NK cells, post-isolation, if required.

#### Collection of NK cells for staining

All work with the NK cell cultures should be carried out in the designated biological safety cabinet using aseptic techniques. Create a homogenous single cell suspension by pipetting up and down. Collect a sample for a cell count. Collect at least 7.5 x 10^4^ cells per condition, as stated below: (i) ‘Unstained Fc block only’ (one control sample), (ii) ‘Viability stain only’ (one control sample), and (iii) ‘All stain’, for each donor to be tested, and fluorescence minus one (FMO) (optional). Label wells of a 96-well plate, or flow cytometry tubes, and transfer the appropriate volume of cell suspension for 7.5 x 10^4^ cells per well/tube.

#### Antibody and viability dye staining of NK cells

Wash cells twice with PBS. Each wash step involves resuspension in 200 µl of either PBS or fluorescence activated cell sorting (FACS) buffer (for 96-well plate) or 500 µl of PBS/FACS buffer (for tubes) followed by centrifugation at 300 x g for 5 minutes. Prepare a 1 in 200 dilution of Zombie Aqua Live/Dead dye in PBS (0.5 µl in 100 µl per test). Re-suspend cells in 100 µl of the pre-diluted viability dye, do not stain the unstained samples or the live/dead FMO samples. Incubate at room temperature (RT) for 15 minutes, protected from light. Following incubation, add PBS to begin washing the cells with centrifugation. Wash cells once again in PBS and centrifuge as before. Prepare a dilution of Fc block at 5 µl/10^6^ cells in FACS buffer (PBS + 1% FBS) (i.e., in this case, 0.4 µl Fc block in 100 µl FACS buffer per condition, as each condition has 7.5 x 10^4^ cells). After wash, discard supernatant and add Fc block to the samples and gently vortex. Incubate at RT for 10 minutes, protected from light. (Note: during this incubation it may be useful to prepare the ‘All stain’ and ‘FMO’ antibody cocktails for cell staining as indicated in the next step). Prepare antibody cocktail in a total volume of 100 µl FACS buffer per sample, as indicated in Table 5 (keep all antibodies on ice). This can be prepared as a master-mix if staining multiple samples at once. Use the table as appropriate to calculate antibody volumes to be added.

**Table 5.** Antibody list for immunophenotyping of natural killer cells by flow cytometry.

Catalog no.	Antibodies/reagents	Volume per sample (µl)	Purpose
BioLegend^®^ #3022303	CD19 PerCp-Cy5.5	5	Exclusion of B cells
BioLegend^®^ #367108	CD14 APC/Cyanine7	2	Exclusion of monocytes and macrophages
BioLegend^®^ #300306	CD3 FITC	3	Exclusion of T cells
BioLegend^®^ #362532	CD56 Brilliant Violet 650	2	NK population identification
BioLegend^®^ #302026	CD16 Alexa Fluor 700	3	NK population identification
BioLegend^®^ #331908	NKp46 PE	2	NK population identification
BioLegend^®^ #422302	Human TruStain FcX	Already added in previous step	Fc blocking reagent
BioLegend^®^ #423102	Zombie Aqua	Already added in previous step	Exclusion of dead cells

For full stain, add all antibodies in mentioned volume to 100 µl FACS buffer and store on ice. For FMO samples, add appropriate volumes of all-but-one antibodies in 100 µl of FACS buffer. Store master mixes on ice away from direct light. All antibody volumes have been determined by titration and calculation of the stain index. However, it might be appropriate to perform titrations depending on sensitivity of flow cytometer used. Here, a BD FACSCelesta™ was used.

**Figure 3:**
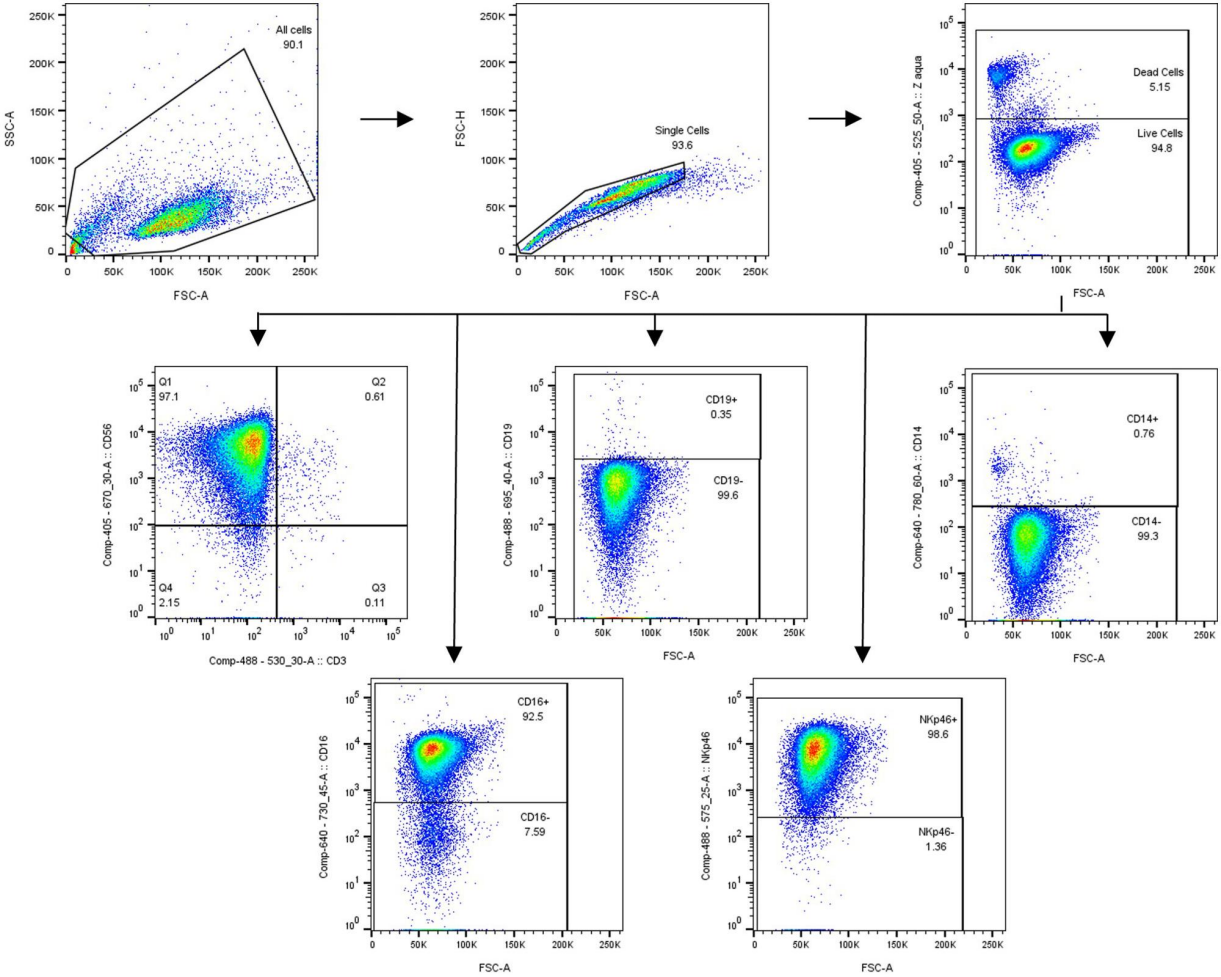
Flow cytometry gating strategy used to measure the viability and immunophenotype of the isolated NK cells following MACS separation. Small debris was initially excluded using FSC-A/SSC-A plot. Cell doublets were then removed using a single cell gate using FSC-A/FSC-H plot. Next, dead cells were excluded using Zombie Aqua viability dye staining. The purity of the NK cell fraction was confirmed by determining CD56 and CD3 positivity, showing a low percentage of CD3-CD56- cells and minimal contamination of CD3+ T cells or CD3+CD56+ NK T-like cells. Minimal contamination from other non-NK cell types was confirmed by measuring CD19 expression to detect B cells and measuring CD14 expression to detect monocytes and macrophages. CD16 and NKp46 expression were used as additional markers to further confirm the isolated population as NK cells

Following incubation with Fc block, wash samples with FACS buffer and centrifuge cells once, as before. Discard supernatant carefully. From this point, all work should be carried out on ice. Re-suspend cell pellet in the corresponding prepared antibody cocktails at 4°C for 30 minutes, protected from light. Following incubation, add FACS buffer and centrifuge cells. Wash in FACS buffer and centrifuge again to remove all unbound antibodies. Finally, re-suspend cells in 200 µl of FACS buffer. Samples are now ready to be acquired on a flow cytometer. A representative result of NK cell viability and purity post isolation is shown in Fig. 3.

### NK cell banking

Biobanking plays a crucial role in the development of cell therapy products by providing high-quality biological samples that are essential for future research and clinical applications. Cryopreservation is known to be harsh on cells due to several detrimental effects that occur during the freezing and thawing processes. This often result in loss of cellular function post-thaw and is a major bottleneck in developing cellular therapies. To prepare a biobank of NK cells, collect NK cells by day 14 in a 50 ml tube. Count cells using a haemocytometer or a cell counter. Centrifuge cells at 300 x g for 5 minutes. Based on cell count, calculate number of cryovials required and determine volume of Cryostor CS10 needed to re-suspend NK cells at a final density of 35 x 10^6^ cells/ml (optimized NK cell freezing concentration, data not shown). Post centrifugation, discard supernatant and re-suspend the cell pellet in Cryostor CS10. Mix gently to form a homogeneous mixture. Quickly aliquot the NK cells into pre-labelled cryovials and place them in a −80°C freezer overnight using cell freezing containers (such as Mr Frosty), for rate-controlled freezing. Transfer the cryovials to liquid nitrogen the following day for long-term storage.

To thaw primary NK cells, pre-warm 10 ml complete NK MACS medium supplemented with 500 IU/ml IL-2 and add 100 units of benzonase in a 15 ml tube. Take an NK cell vial from the liquid nitrogen and quickly thaw in the water bath. Transfer the cells from the cryovial to the 15 ml prepared tube. Centrifuge cells at 300 x g for 5 minutes and discard supernatant. Add NK MACS complete medium. Depending on number of cells thawed, transfer cell suspension to T25/T75 flask and top-up flask with additional medium to maintain a seeding density of 5 x 10^5^ cells/ml. Supplement medium with 500 IU/ml IL-2. Transfer flasks to incubator. The NK cells are ready for experimental use within 24 hours.

### NK cell functionality assessment

Assessing the cytotoxic potential of the NK cells is undoubtedly the most crucial step for optimizing any translational research. To measure the functionality of these *ex vivo* expanded NK cells, use a 20 h co-culture assay of NK cells with the cancer cells (K562 cells, in this case). Prior to assay setup, warm up complete RPMI and complete NK MACS medium. Label K562 cells with a cell tracking dye (Tag-IT violet) using the manufacturer’s protocol. Count the cells and seed them in a 96-well plate at 20,000 cells in 100 µl complete RPMI medium per well. During NK cell preparation, leave the 96-well plates in a cell culture incubator. Calculate total number of NK cells required for assay setup. The final volume of each well should be 200 µl. Count NK cells and collect appropriate volume of cell suspension for required NK cell numbers in a 50 ml tube. Centrifuge NK cells at 300 x g for 5 minutes and discard supernatant. Re-suspend NK cells in fresh complete NK MACS medium supplemented 500 IU/ml IL-2. Take the 96-well plate out of the incubator and into the biological cabinet. Gently mix the NK cells to form a homogenous suspension and add calculated volume of NK cell suspension to attain desired effector to target (E: T) ratios in appropriate wells. For example, at 1:1 E: T, seed 20,000 NK cells in 100 µl NK MACS +IL-2 medium against 20,000 target cells in 100 µl complete RPMI medium. If required, top up wells with 500 IU/ml IL-2 supplemented NK MACS medium to bring the final volume in each well to 200 µl. Consider adding the following control wells—E:T 1:0 and 0:1 for further cytotoxicity calculations.

Once assay is set up, transfer plates to 37°C incubator for 20 h. Prepare propidium iodide (PI) dead cell staining solution in PBS to a concentration of 100 µg/ml, and store in 4°C fridge, protected from light. After 20 h, collect the 96-well plate, add 1 µl of the PI solution to each well and acquire samples on flow cytometer (in case of large number of samples, store plates on ice and add PI immediately before acquisition on flow cytometer). On the flow cytometer, gate the Tag-IT^+^PI^+^ cells (dead target cells) from the parental ‘all events’ gate.

Calculate specific NK cell cytotoxicity using the formula:

[(Experimental well target cell death %)—(background target death %)]/[100—(background target death %)] x 100%, where background target death indicates the percentage of target cell death in 0:1 E:T wells.

### Potential issues and mitigation

Collecting the peripheral blood mononuclear cell (PBMC) layer after Ficoll separation can be difficult for inexperienced operators. This step can be made easier by the use of SepMate tubes. The initial PBMC cell counting step prior to NK enrichment can be challenging due to platelet contamination or potentially high PBMC numbers from some donors. Users can dilute the PBMCs for counting as needed and/or do a 10-fold dilution of the cell suspension with 3% acetic acid with methylene blue solution (07060, StemCell Technologies) to lyse red blood cells and platelets. Subsequent counting during the initial days of expansion (day 4, day 6) can be complicated by the presence of debris from dying feeder cells. Counting confidence can be improved by using a viability dye such as Trypan Blue or Erythrosine B. Smaller MS separation columns are also available, however for the cell numbers and volumes outlined here, we have found that the MS columns can become clogged preventing cell flow through, thus the current protocol recommends the LS columns. Nonetheless, the MS columns can be useful if smaller starting volumes of blood are used. For immunophenotyping, it is important to use reliable and titrated antibodies. Fc blocking should also be employed to prevent false positive reading by antibodies binding to Fc receptors expressed by various immune cells.

## Results

The isolation of NK cells from peripheral blood is a critical first step for developing and testing any NK-based cellular immunotherapies. A graphical representation of the optimized isolation protocol workflow is shown in Fig. 1 and a detailed video demonstrating the steps for isolation is available as Supplementary data, Video 1.

MACS has been shown to be an effective method for NK cell isolation from PBMCs. The negative selection technique utilizes antibody labelling against a wide range of hematopoietic cell surface markers that are not present on NK cells, which are then conjugated with superparamagnetic microbeads. The magnetically tagged non-NK cells are retained in the separation column placed in a magnetic field, while the non-tagged NK cells pass through the column untouched and are collected in the flow-through fraction, resulting in an enriched NK cell population. Figure 2 shows representative microscopy images taken before (Fig. 2A) and after (Fig. 2B) magnetic sorting of the PBMCs. This isolation protocol yields an average of 3.1 x 10^6^ NK cells from 25 ml of peripheral blood, which equates to an average proportion of 9.5% NK cells from the PBMC population (*n* = 20), (Fig. 2C).

The isolation process does not cause severe stress to the NK cells, as the isolated cells retain over 90% viability. Enrichment and purity of the NK cell fraction can be determined with the optimized immunophenotyping assay designed to measure the amount of contaminating B cells (CD19^+^), T cells (CD3^+^), monocytes and macrophages (CD14^+^) present in the flow-through sample (NK cell fraction), where the NK cell population is identified as the CD3^-^/CD56^+^ fraction. Additionally, NKp46 and CD16 were used to confirm the identity of the NK cell population. Results confirm that the NK cell fraction is of high purity as the CD19^+^, CD3^+^, CD14^+^ populations were all negligible, less than 1% of the total live cell population, and close to 99% of the isolated cells were CD3^-^CD56^+^ and positive for NKp46, and over 90% was also positive for CD16. Representative flow cytometry data and gating strategy for measuring NK cell viability and immunophenotype from one donor, post-isolation (day 0) is shown in Fig. 3.

**Figure 4:**
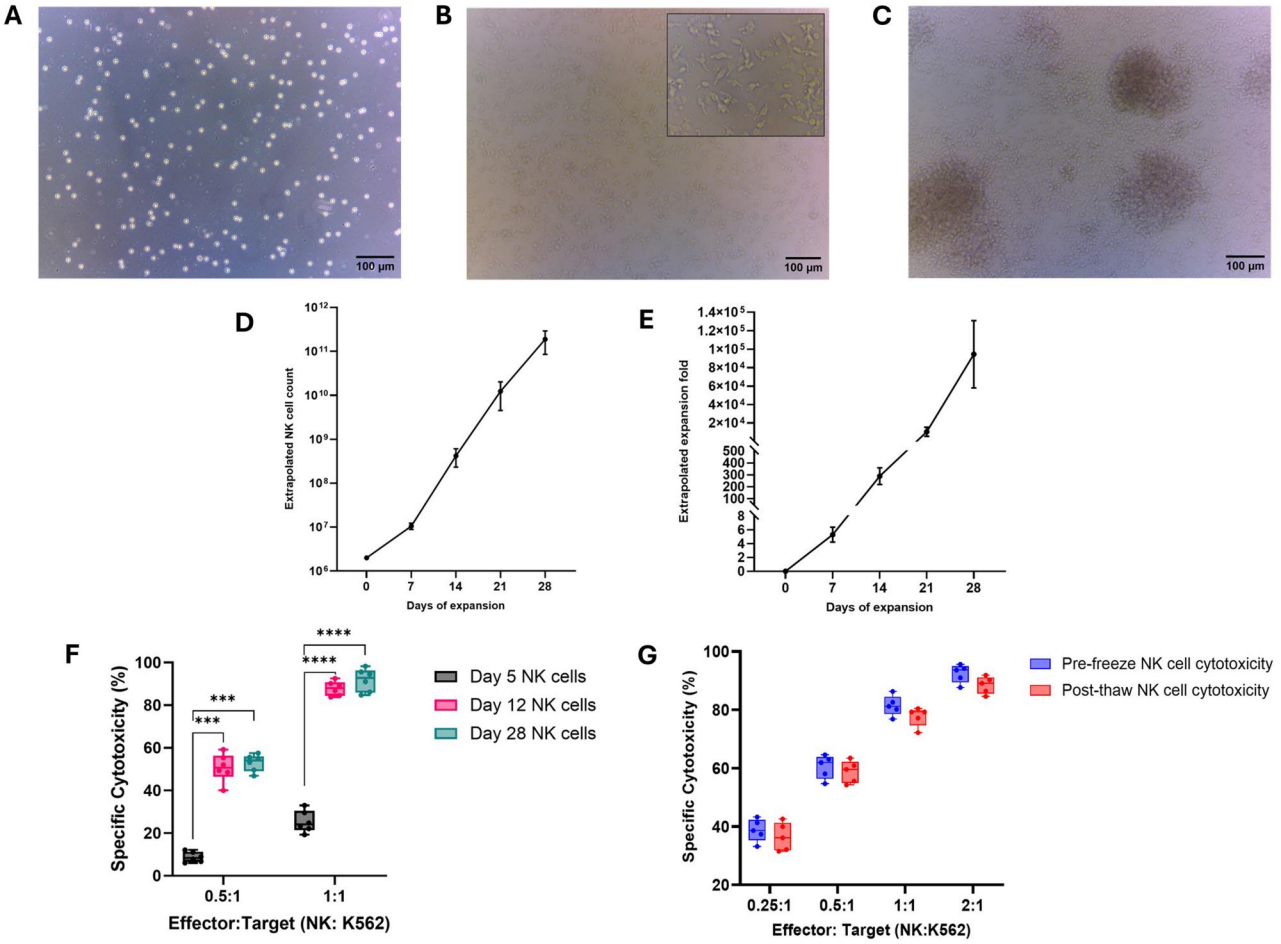
Typical NK cell morphology, expansion, and confirmation of cytotoxic capability. (A) Morphology of the NK cells on day zero of isolation at 100x magnification. (B) Morphology of NK cells during expansion on day seven post isolation at 100x magnification. Inlaid image is at 200x magnification. (C) Upon stimulation by cytokines, NK cells form clumps by expansion day seven. Image was taken at 100x magnification. All scale bars show 100 μm. (D) On day zero, NK cells were stimulated with irradiated EBV-LCL feeder cells (NK:Feeder 1:10) and were supplemented with 100 ng/ml IL-21 and 500 IU/ml IL-2. The same concentration of IL-2 was replenished every 48 h. IL-21 was added on day zero only. Extrapolated cell counts of NK cells in culture are shown (*n* = 10). Cells were counted and sub-cultured every 48 h. (E) Extrapolated cell counts were used to calculate cumulative fold change in the NK cell populations (*n* = 10). (F) Expanded NK cells were co-cultured with K562 cell line at different effector to target cell ratios for 20 hours. Cell death was then determined using PI live/dead staining and measured by flow cytometry. The *ex vivo* expanded NK cells exhibit high (*P* ≤ 0.0005) and consistent cytotoxicity against K562 target cells in vitro. Cytotoxicity is maintained from day 12 to day 28 of expansion. (G) Comparison of NK cell functionality pre-freeze and post-thaw against K562 target cells. NK cells from day 14 of expansion were subjected to a 20 h co-culture assay with the K562 cells. Stocks of same donor derived NK cells, frozen on day 14 of expansion were thawed and subjected to 20 h co-culture assay with K562 cells, following a brief 4 h recovery in complete NK MACs media supplemented with 500 IU/ml IL-2. NK cells retained their functionality post thaw (*P* ≥ 0.05)

Morphologically, the NK cells appear more spherical on day 0 (Fig. 4A). By day seven, they take on a lymphoblastoid, irregular shape with a large nucleus (Fig. 4B). Upon cytokine stimulation, they typically start forming clusters/loose clumps which can be easily broken up by gentle pipetting (Fig. 4C). *Ex vivo* expansion of the NK cells isolated using this protocol consistently generates robust NK cell numbers. Upon seeding 2 x 10^6^ NK cells on Day 0, an average of 1 x 10^7^ cells can be harvested on day 7, 4.8 x 10^8^ cells by day 14, 1.24 x 10^10^ cells by day 21, and 1.89 x 10^11^ cells by day 28 (Fig. 4D), achieving an average 1.04 x 10^4^ fold change by day 21 and 9.45 x 10^4^ cumulative fold change by day 28 (*n* = 10) (Fig. 4E). Functionally, the NK cells demonstrate a low level of cytotoxicity by day 5 of expansion. This is significantly enhanced by day 12 and is maintained throughout until at least day 28 of their expansion. This was determined by measuring NK-mediated killing of the target K562 cells using a straightforward co-culture assay where the target cell fraction was co-cultured with the effector NK cells at different effector to target ratios, for 20 h. The graph in Fig. 4F compares *ex vivo* expanded NK cell cytotoxicity at day 5 vs day 12 vs day 28 of expansion, at an effector to target ratio of 0.5:1 and 1:1 (*n* = 6). Finally, to show that cryopreservation did not diminish NK cell functionality, the cytotoxic potential of the NK cells before and after cryopreservation from the same donor of NK cells were checked using the same 20 h co-culture cytotoxicity assay as above. Results indicated that the NK cells expanded and frozen using this protocol retained comparable levels of cytotoxicity (*n* = 5) (Fig. 4G).

## Discussion

This protocol describes a robust method for isolating NK cells from human peripheral blood samples well-suited for translational research for NK cell-based cellular immunotherapies. The results demonstrate that negative, magnetic-activated cell sorting provides high yield and purity of NK cells. These cells can then be expanded *ex vivo* to attain clinically relevant numbers of NK cells characterized by high functionality.

The NK cells isolated here maintained high viability and consisted of a highly pure cell population with negligible contamination by B cells, T cells, and monocytes/macrophages, comparable with other studies [29–31]. The contamination of other haematopoietic cell types in NK cell preparations during *ex vivo* expansion poses significant challenges to the efficacy and safety of NK cell-based immunotherapies. T cells can expand significantly during culture, thereby compromising the purity and functionality of a potential NK cell product [32]. The presence of T cells can also lead to GvHD after patient infusion [33]. B cell contamination in NK cell preparations has the potential to cause haemolytic anaemia mediated by the donor passenger B cells producing antibodies against the recipient’s red blood cells [34, 35]. Contamination from monocytes/macrophages can potentially exacerbate inflammatory responses and lead to cytokine-related toxicities [36].

Although magnetic sorting of cells is widely used to isolate various cell types including NK cells, it has been associated with potential functional impairment of the NK cells due to antibody cross-linking [15, 37]. Additionally, positive selection may only isolate specific sub-populations of NK cells, potentially missing the diverse repertoire of NK cell subsets [15]. The negative selection technique, or the ‘untouched’ approach using microbead-coupled antibodies against a wide range of non-NK haematopoietic cell surface markers allows for an improved and enriched NK cell yield, resulting in a better viability and potent *ex vivo* proliferation. Furthermore, negative selection results in the isolation of a more heterogeneous population of haematopoietic cells, including less differentiated subsets of NK cells, that may have greater downstream differentiation and proliferative potential, to pass through the column while removing all other common blood cell types [15, 38].

Regardless of the isolation method, the expansion and activation of the isolated NK cells is a critical second step for generating clinically relevant numbers of cells for adoptive therapy. Appropriate NK cell dosage may vary depending on the specific clinical setting, the patient’s disease status, the method of NK cell expansion and activation, along with the purity and functional status of the infused NK cells. In the context of haematological malignancies, such as acute myeloid leukaemia and multiple myeloma, clinical trial studies have reported the use of NK cell doses ranging from 5 × 10^6^–1 × 10^8^ cells/kg [39, 40]. For solid tumours, such as hepatocellular carcinoma and lung cancer, the reported NK cell doses have been in the range of 1 × 10^9^–3 × 10^10^ cells per infusion [41, 42]. The expansion protocol outlined here was able to produce an average of 1.9 x 10^11^ NK cells from as little as 25 ml of blood, making it suitable for scale up to a clinical manufacturing level, considering 10^6^–10^10^ cells per kilogram of patient weight in a single infusion [31, 39–42]. The use of feeder cells, such as EBV-LCL which naturally express the ligand for 4-1BB, a stimulatory receptor on NK cells, in combination with the cytokines IL-21 and IL-2, has been shown to be a highly efficient approach for *ex vivo* expansion of human NK cells with high anti-tumour activity [21, 43]. While there has also been success with expanding NK cells using non-feeder cell-based approaches, the fold expansion achieved was limited [10], in the range of 5–30 fold with IL-2 and 175-fold using IL-2 together with IL-18 [23, 18, 44]. In addition to the EBV-LCL feeder cells, which originated from the Child’s laboratory, several magnitude NK cell expansion has been demonstrated with a range of feeder cells, including K562 cells engineered to express membrane bound IL-21 or IL-15 [20, 45]. Another source of feeder cells could be commercially available EBV transformed LCL cells, although these have not been tested by our group.

These expanded NK cells exhibited potent cytotoxicity, against K562 cells, which was consistent from day 12 to day 28 of expansion. This agrees with previous reports demonstrating the ability of *ex vivo* expanded NK cells to effectively kill primary acute lymphoblastic leukaemia cells and multiple myeloma cells [46]. Although this protocol shows that a single dose of IL-21 and continuous stimulation by IL-2 is sufficient to drive NK cell proliferation and maintain their cytotoxicity, we have not tested the immunophenotype or functionality of the NK cells in an *in vivo* setting, post *ex vivo* expansion.

This protocol has been designed with GMP translation in mind. The use of animal-derived reagents has been avoided with our choice of hAB serum during cell culture and xeno-free NK MACS medium. Furthermore, antimicrobials are not used during cell culture. The EBV-LCL feeder cell line utilized here has been GMP certified and has been used in the clinic [47]. GMP grade IL-2 and IL-21 stimulatory cytokines are also available (Miltenyi Biotech) to further support the clinical translation of this method. As noted in the section ‘NK cell seeding and feeder cell addition’, the method can also be expanded for larger volume using for example the G-Rex system, which can be upscaled to a certified cell manufacturing facility with grade A clean rooms. The expansion process could further adhere to high GMP standards by potentially transitioning to a closed G-rex culture system [47, 48]. Extensive validation of cell immunophenotyping by flow cytometry and proof of NK cell function with initial *in vitro* cytotoxicity assays have been outlined here which can provide a basis for designing quality control assays for product purity and potency [49].

Engineering NK cells has become an area of active and ongoing research in order to improve their *in vivo* persistence and cytotoxicity. Building on from the success of CAR-T cell therapy, NK cells can also be engineered to express CARs to enhance their tumour targeting capabilities [17, 50]. The method outlined here has been successfully used by our group to obtain and expand NK cells that were then further manipulated by CD38 knockdown and the addition of a CD38 CAR [51].

Overall, these results demonstrate that a MACS-based isolation protocol, coupled with the feeder cell-based expansion method with a minimalistic cytokine stimulation is sufficient to generate clinically relevant numbers of highly pure and functional NK cells. This generates NK cells suitable for a range of research purposes and with an outlook to clinical translation and the development of effective NK cell-based immunotherapies.

## Supplementary Material

bpaf015_Supplementary_Data

## Data Availability

The data underlying this paper are available in the article and in its online supplementary material.
