# Supplementary figures and images for: Protocol for isolation and expansion of natural killer cells from human peripheral blood scalable for clinical applications
Source: Biol Methods Protoc. 2025 Feb 26;10(1):bpaf015. doi: 10.1093/biomethods/bpaf015 (PMC11889455; doi:10.1093/biomethods/bpaf015)

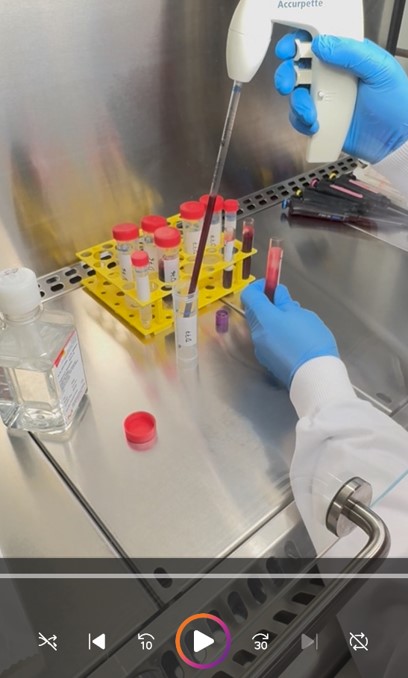

Supplement: bpaf015_Supplementary_Data [file bpaf015_supplementary_data.zip › Video_1_still_image.jpg]
